# A comparison of inpatient with outpatient balloon catheter cervical ripening: a pilot randomized controlled trial

**DOI:** 10.1186/s12884-015-0550-z

**Published:** 2015-05-28

**Authors:** Chris Wilkinson, Pamela Adelson, Deborah Turnbull

**Affiliations:** Maternal-Fetal Medicine, Women’s and Children’s Hospital, Hospital, 72 King William Road, North Adelaide, SA Australia; School of Psychology, University of Adelaide, Adelaide, SA Australia

**Keywords:** Cervical ripening, Induction of labour, Outpatient ripening, Double balloon catheter

## Abstract

**Background:**

One in four Australian births are induced. If cervical ripening using a prostaglandin is required, a pre-labour overnight hospitalisation and separation from family and support companions is necessary. Recent evidence shows that balloon catheter cervical ripening is just as effective as prostaglandins, but does not cause uterine stimulation. For women with low risk pregnancies, this offers the possibility of undergoing the overnight ripening process in their own home. We conducted a pilot randomised trial to assess the outcomes, clinical pathways and acceptability to both women and clinicians of outpatient balloon catheter ripening compared with usual inpatient care.

**Methods:**

Forty-eight women with low risk term pregnancies were randomised (2:1) to either outpatient (*n* = 33) or inpatient double-balloon catheter (*n* = 15) cervical ripening. Although not powered for statistically significant differences, the study explored potential direction of effect for key clinical outcomes such as oxytocin use, caesarean section and morbidities. Feedback on acceptability was sought from women at catheter insertion and 4 weeks after the birth, and from midwives and doctors, at the end of the study.

**Results:**

Clinical and perinatal outcomes were similar. Most women required oxytocin (77 %). The outpatient group were 24 % less likely to require oxytocin (risk difference −23.6 %, 95 % CI −43.8 to −3.5). There were no failed inductions, infections or uterine hyperstimulation attributable to the catheter in either group. Most women in both groups reported discomfort with insertion and wearing the catheter, but were equally satisfied with their care and felt the baby was safe (91 % both groups). Outpatient women reported feeling less isolated or emotionally alone. Most midwives and doctors (*n* = 90) agreed that they are more comfortable in sending home a woman with a catheter than prostaglandins and 90 % supported offering outpatient ripening to eligible women.

**Conclusions:**

Outpatient balloon catheter ripening should be further investigated as an option for women in an adequately powered randomised trial.

**Trial registration:**

Prospectively registered, Australian New Zealand Clinical Trials Registry ACTRN12612001184864.

## Background

Induction of labour is undertaken in about a quarter (26 %) of all births in Australia, with the main reasons being for prolonged pregnancy or psychosocial reasons [1]. For women who have an unfavourable cervix for labour induction, the induction process often begins with a pharmacological cervical ripening agent such as prostaglandin E2. These preparations are convenient to use and quick acting in bringing about cervical ripening. However, they are costly, involve a pre-labour overnight hospitalization and can have undesirable side effects for both mother and baby, particularly from hyperstimulation. An alternative is the mechanical method of transcervical balloon catheter ripening, a technique that was first used decades ago and has recently experienced an international resurgence as a method that is efficacious, has fewer side effects, and may be less costly than pharmaceutical methods. Recent studies have validated the use of balloon catheters and found them to be equally effective and safe as pharmacological methods, with the advantage of not causing hyperstimulation and unfavourable cardiotocograph (CTG) changes [2–4].

Most balloon catheter ripening is done as an overnight hospitalisation along the same protocols as pharmacological methods. However the lack of direct myometrial stimulation may reduce the need for close and continuous monitoring of mother and baby in a hospital environment and existing evidence suggests that that balloon catheters may have a role in the outpatient setting. A trial of 111 women published in 2001 comparing single balloon catheter outpatient ripening with inpatient single balloon catheter ripening foundthe outpatient approach to be just as effective, with no difference in clinical outcomes or adverse events [5]. A Cochrane review of outpatient induction of labour concluded that outpatient induction is feasible and that important adverse events are rare. In addition, it concluded that there was insufficient evidence to know which induction methods are preferred by women, or which interventions are the most effective and safe to use in outpatient settings [6].

In 2008–11 we conducted an outpatient ripening trial, the OPRA Study, with 827 low risk women comparing outpatient and inpatient cervical ripening with prostaglandin E2. We found no significant differences in maternal or neonatal outcomes [7], and improved psychosocial outcomes [8]. Women preferred outpatient care and did not experience additional anxiety upon randomisation to the outpatient group. Economic evaluation was favourable for outpatient care [9], and the practice was supported by midwives [10]. However, we did find that because of uterine stimulation or non-reassuring fetal monitoring following prostaglandin administration, less than half of the women who intended to go home overnight actually went home or remained home overnight. Thus, planning to discharge women for a night’s rest after prostaglandin cervical ripening does not achieve that aim for the majority of women. We also found that pregnancy complications (hyperstimulation and hypoxaemic ischaemic encephalopathy) were more than expected for a low risk population for both inpatient and outpatient prostaglandins ripening.

Our experience with the OPRA Trial found that although the practice of outpatient ripening was supported, prostaglandins might not be the best vehicle by which to achieve this. Subsequently, we conducted an exploratory pilot trial to explore the need for a larger randomized controlled trial with balloon catheter ripening. The objectives of this pilot were to (1) To compare key labour and birth outcomes in inpatient compared with outpatient catheter ripening for direction of effect and magnitude (2) To assess the clinical pathways of the intervention and determine the acceptability from the perspective of both pregnant women and health care providers.

## Methods

We conducted a pilot randomized controlled trial at the largest maternity teaching hospital (Women’s and Children’s Hospital) in Adelaide, South Australia, between October 2012 to July 2013, after obtaining approval from the Womens and Childrens Health Network Human Research Ethics Committee (REC2453/3/15). A designated research midwife, not involved in patient care, recruited eligible women scheduled for induction who were attending public clinics or the birth centre. Written, informed consent was obtained and documented. Randomisation was delayed until just after the catheter was inserted, so that women randomised to an intervention were most likely to receive that intervention. Eligible women were randomized to either staying in hospital or to go home for cervical ripening with a double-balloon cervical ripening catheter (Cooks® Cervical Ripening Balloon J-CRBS-184000). Eligibility criteria included: term (37–42 weeks), healthy pregnancy; intact membranes and bishop score of < 7; singleton, cephalic presentation and appropriately grown; cervical ripening being done for reasons other than fetal or maternal compromise (i.e., low risk, post dates and social inductions, excluding previous caesarean sections).

### Randomization and analysis

Randomization was stratified for parity and a 2:1 (outpatient to inpatient) ratio was used in order to maximize our experience with outpatient management. A computer-generated list with randomly allocated block sizes was prepared and sequentially numbered. Allocation assignments were placed and sealed in opaque envelopes by a person not otherwise involved in the conduct of the trial and were securely held in the area where randomization occurred. Envelopes were only opened after participant details were recorded.

The pilot study was not powered to determine statistically significant differences in clinical or psychosocial outcomes, but to determine potential direction and magnitude of effect. Analysis was by intention to treat and data analysis was performed with SPSS and EpiInfo [11, 12]. As this was a pilot study, analysis was primarily descriptive and differences focused on confidence interval estimations [13]. Clinical and system data were directly collected and entered into a computer database by the designated research midwife. Data were independently cross-checked and reviewed for accuracy.

Acceptability of the alternative priming processes was measured at catheter insertion and four weeks after delivery. Immediately following catheter insertion women were asked to indicate on a visual analogue scale the degree of discomfort they felt by marking on a 10 cm line, with zero at the far left of the scale (no discomfort) to 10 cm at the far right (extreme discomfort). Four weeks after the birth, a de-identified questionnaire similar to the validated instrument used in the OPRA trial was mailed to women, with a two week follow-up for non-responders. The questionnaire sought information on satisfaction with care, preparedness, the induction environment and specific items relating to the catheter ripening process. Responses were organized in a 5-point Likert scale response format. Two additional free text response questions asked women their feelings on having the catheter in place and positive and negative aspects of this method.

### Cervical ripening protocol

Eligible participants presented to hospital in the afternoon for ripening and underwent 20 min of pre-catheter CTG monitoring. Following satisfactory monitoring, a clinician (doctor or midwife trained to insert the catheter) inserted a double balloon catheter and inflated each balloon with 70–80 mls of sterile water in the Women’s Assessment Service (the hospital’s emergency department). Women were subsequently randomized to inpatient or outpatient care and CTG monitoring was maintained for a minimum of 20 min post insertion.

Those randomized to outpatient care were discharged home following satisfactory CTG monitoring with written instructions and a direct telephone number to the senior midwife on duty in the Women’s Assessment Department. The woman was requested to remain at home and to return to the labour ward at 08.00 the following morning or earlier in the event of onset of labour, rupture of membranes, vaginal bleeding or other complications or concerns. Women remained at home until the following morning in the absence of labour onset or if the catheter fell out.

The pathway for both inpatient and outpatient women was the same the following morning; An amniotomy was performed, followed by an oxytocin infusion if labour did not begin within 4 h, in accordance with the South Australian Perinatal Practice Guidelines on induction of labour. (http://www.sahealth.sa.gov.au/wps/wcm/connect/public+content/sa+health+internet/clinical+resources/clinical+topics/perinatal+practice+guidelines/perinatal+practice+guidelines).

### Clinician survey

Catheter priming was a novel intervention at the investigating institution. Orientation seminars were conducted prior to the study and individual training given by senior staff as needed. In order to determine clinicians’ views of this new method, an anonymous questionnaire was distributed to both doctors and midwives who were involved in the ripening process at the conclusion of the pilot study. The questionnaire sought information on the clinical experience and opinions of the catheter in a 5-point Likert scale response format. The questions were modelled on survey instruments used by the authors in similar published work [10]. Additional questions were formatted as free text responses exploring opinions of catheter ripening and views of what should be considered for outpatient ripening to be a sustainable practice.

## Results

**Fig. 1:**
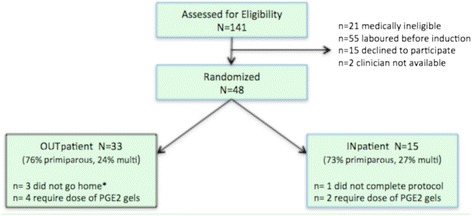
Recruitment and randomization into COPRA study. *1 change her mind & remained in hospital, 1 small bleed after insertion, 1 unsatisfactory CTG during post-insertion monitoring

A total of 141 women were assessed for eligibility, of whom a large proportion spontaneously laboured before the scheduled induction. Of the remaining potentially eligible 63 women, fifteen (24 %) declined to participate. Three women were not randomized due to catheter associated issues: clinician unable to insert catheter, spontaneous rupture of membranes on balloon inflation, and fetal heart rate deceleration noted at insertion. All three were admitted and managed without complications. This left 48 women who were randomized into the study; 33 outpatients and 15 inpatients (Fig. 1).

### Clinical outcomes

The characteristics of the two groups were similar with the primary reason (90 %) for induction of labour being post-dates or to avoid prolonged pregnancy (Table 1).

**Table 1:** COPRA characteristics (n = 48)

Characteristic^a^	Inpatient *N* = 15 (%)	Outpatient *N* = 33 (%)
Parity		
Nulliparous	11 (73.3)	25 (75.7)
Parous	4 (26.6)	8 (24.2)
Mean (SD) age	29.1 (6.8)	28.9 (4.2)
Marital status		
Married/Defacto	12 (80.0)	32 (96.8)
Single	3 (20.0)	1 (3.1)
Language spoken at home		
English only	13 (86.7)	24 (75.0)
Other language	2 (13.3)	8 (25.0)
Education		
University degree	5 (35.7)	17 (53.1)
Post-high school training	6 (42.9)	11 (34.4)
High school only	3 (21.4)	4 (12.5)
Reason for induction		
Prolonged pregnancy	13 (86.7)	26 (78.8)
To avoid prolonged pregnancy	-	4 (12.1)
Social	-	1 (3.0)
Other^b^	2 (13.3)	2 (6.1)
Gestation at priming Median weeks^+ days^ (IQR)	40^+8^ (40^+5^- 40^+10^)	40^+7^ (40^+6^- 40^+10^)
Modified Bishop’sscore		
0–2	5 (33.3)	8 (25.8)
3–4	9 (60.0)	16 (51.6)
≥5	1 (6.6)	7 (22.6)
Mean (SD) time waiting for catheter insertion (range 15 mins to 5 h)	1 h 36 mins (1 h 09 mins)	2 h 02 mins (1 h 21 mins)
Mean (SD) time waiting for catheter to discharge from Women’s Assessment service	2 h 55 mins (1 h 02 mins)	3 h 02 mins (1 h 26 mins)
Catheter insertion technique		
Speculum	13 (92.9)	25 (78.1)
Digital	1 (7.1)	7 (21.9)
Mean (SD) pain score insertion *(scale 0–100 visual analogue scale)*	25.1 (20.3)	31.1 (24.8)
Catheter removal		
Spontaneously fell out in hospital	2^c^ (13.3)	1^d^ (3.0)
Spontaneously fell out at home	n/a	11^e^ (33.3)
Clinician removed	13 (86.7)	21 (63.6)
Mean (SD) time catheter in situ (removed by clinician)	14 h 48 min (4 h 5 min)	14 h 32 min (2 h 22 min)
Mean (SD) time catheter spontaneous fell out	6 h 35 mins (5 h 18 mins)	10 h 50 min (4 h 13 mins)
Mean (SD) length of time at home with catheter	n/a	12 h 27 mins (2 h 50 mins)
Outpatient women return to hospital before scheduled	n/a	2 (6.7 %)^f^

Catheters spontaneously fell out in 29.2 % of all women (14/48), in 66.7 % of all parous women (8/12), and in 16.7 % of all nulliparous women (6/36)

*n* = 1 contraction onset, returned 3:45 am

^a^Maximum amount of not stated for any variable n=2 (Bishop score, VAS pain score, insertion technique)

^b^Other reasons= back pain, previous term stillbirth, previous large baby

^c^Both women were multiparous

^d^Woman was primiparous

^e^n=6 women were multiparous

^f^Based on n=30 discharged home. Reason for return; n=1 anxious, returned 10 pm

The labour and delivery outcomes for the two groups were also similar (Table 2). Most women required oxytocin (77 %) either for induction or augmentation of labour. Women who had outpatient ripening were about 24 % less likely to require oxytocin (risk difference of −23.6 %, 95 % CI −43.8 to −3.5) and had a lower caesarean section rate, although this was not statistically significant (risk difference of −15.1 %, 95 % CI −42.4 to 12.1). There were no cases of failed inductions, infections or uterine hyperstimulation attributed to the ripening catheter. Six nulliparous women (12.5 %) in the study also required a single dose of PGE_2_ gels as amniotomy was not possible after catheter removal. Half of these women eventually required a caesarean section. The overall caesarean section rate was 23 %, a rate similar to that of women (25 %) who received PGE_2_ ripening in our recently published outpatient prostaglandin Trial (OPRA) [7].

**Table 2:** COPRA labor and delivery outcomes (N = 48)

Variable	Inpatient *N* = 15 (%)	Outpatient *N* = 33 (%)	Risk difference (95 % CI)
Rupture of Membranes			
Spontaneous	0	4 (12.1)^a^	-
Artificial Rupture of Membranes	15 (100)	30 (90.9)^b^	
Oxytocin Infusion	14 (93.3)	23 (69.7)	−23.6 % (−43.8 to −3.5)
Reason for oxytocin infusion			
Induction oflabor	8 (57.1)	14 (60.9)	
Augmentation	6 (42.8)	9 (39.1)	
Duration of oxytocin infusion	5 h 9 mins	7 h 47 mins	
Mean (SDminutes)	(3 h 51 mins)	(4 h 59 mins)	
PGE_2_ given in addition to catheter (ie not ARMable after removal, 1 dose only)	2 (13.3)	4 (12.1)	
Method of delivery			
Spontaneous vaginal	7 (46.7)	16 (48.5)	Overall LSCS rate 22.9 %
Instrumental	3 (20.0)	11 (33.3)	−15.1 % (−42.4 to 12.1)
Caesarean section	5 (33.3)	6 (18.2)	c/s
Indications for caesarean section	(*n* = 5)	(*n* = 6)	-
Fetal distress	1 (20.0)	-	
Lack of progress	3 (60.0)	6 (100.0)	
Other (malpresentation, etc.)	1 (20.0)	-	
Mean (SD) length of active labor, vaginal births	(*n* = 10) 8 h 0 mins (6 h 54 min)	(*n* = 27) 7 h 34 min (4 h 14 min)	
Vaginal delivery within 24 h of priming catheter?	4 (26.7)	11 (33.3)	-
Mean (SD) time catheter inserted to active labour (vaginal delivery)	19 h 49 mins (3 h 3 mins)	17 h 28 mins (3 h 54 mins)	
Mean (SD) time catheter inserted to ARM	17 h 39 mins (5 h 6 mins)	17 h 37 mins (3 h 38 mins)	
Mean (SD) time catheter inserted to vaginal delivery^c^	29 h 01 mins (8 h 5 mins)	24 h 51 mins (5 h 32 mins)	
Mean (SD) hours hospital admission to delivery^c^	21 h 27 mins (5 h 18 mins)	14 h 15 mins (7 h 20 mins)	
Labor analgesiaepidural^d^	11 (73.3)	23 (69.7)	-
Labor complications^e^			
Meconium-stained liquor	1 (6.7)	4 (12.1)	
PPH >500 ml (vaginal births) or 1 L c/s	2 (13.3)	6 (18.1)	
Pyrexia during labor	0 -	1 (3.0)	
Hyperstimulation	1	1^f^	
Failed primings	-	-^g^	

^a^All cases of spontaneous rupture of membranes occurred in hospital

^b^ *n* = 1 case of spontaneous rupture of membranes, followed by ARM

^c^Excludes *n* = 1 outpatient case whose management changed & delivered spont 5 days after catheter removal

^d^Includes *n* = 2 (1 in each group of spinal anaesthesia)

^e^Women may have more than one labor complication. PPH, post-partum hemorrhage

^f^Woman also received PGE_2_ priming gels

^g^ *n* = 1 change of management; women 39^+3^ had catheter for 12 h, high head, IOL abandoned, NVD 5 days later

There were no serious maternal or neonatal morbidities attributed to catheter ripening, as per Table 3. One baby, delivered by caesarean section for fetal distress and failure to progress 14 h post readmission after outpatient priming, was admitted to intensive care following meconium aspiration syndrome. The baby was discharged from hospital in good condition after 17 days. Three babies, (one randomized to inpatient and two to outpatient) were noted as being febrile or given antibiotics. These were individually reviewed and complete blood count and blood cultures showed no lymphocytosis or definitive evidence of infection.

**Table 3:** Neonatal outcomes

Characteristic	Inpatient *N* = 15 (%)	Outpatient *N* = 33 (%)
Gender		
Male	9 (60.0)	18 (54.5)
Female	6 (40.0)	15 (45.5)
Birth weight mean g, (SD)	3721 (522)	3537 (494)
Congenital anomalies	1	-
Apgar <7 at 5 min	-	2^a^ (6.1)
Admission to neonatal intensive care	-	1 (3.0)
Special care nursery admissions:	7 (46.7)	12 (36.4)
Feeding problems	-	1
Respiratory problems	-	1
Blood sugar regulation	-	1
Febrile or antibiotics^b^	2	1
Maternal care	2	2
Other (observation)	3	6
Length of staymean days (SD)	3.9 (1.8)	3.3^c^ (1.3)

^a^One case of meconium aspiration syndrome, baby admitted to neonatal intensive care. The other case involved a tight nuchal cord

^b^Complete blood count and blood cultures showed no growth or signs of infection in babies. In one case mother was febrile after prolonged labour, mother afebrile in other cases

^c^excludes *n* = 1 NICU admission for 17 days unrelated to priming

### Clinical pathway

Catheter insertion (with or without using a speculum) varied according to the clinician’s preference; most were done with a speculum (Table 1). There were 7 minor catheter related occurrences in the 48 women such as vaso-vagal reaction to insertion or altered sensation while voiding. In all cases the issue resolved without complications and the catheters remained in situ overnight. There were two cases of hyperstimulation in the study. Both occurred while an oxytocin infusion was being used and were not attributed to the catheter.

Of the 33 women randomized to outpatient care, three did not go home overnight (Fig. 1). For the 30 outpatient women who were discharged home overnight, only two returned before planned admission the following morning; one returned due to anxiety, the other due to onset of contractions. Catheters fell out spontaneously in 29 % (14/48) of all women either at home or in the hospital and were more likely to fall out in multiparous women (67 % 8/12) than in nulliparous women (17 % 6/36). Outpatient women spent an average of 12.3 h out of hospital after having the catheter inserted.

### Acceptability of the catheter to women

Visual analogue pain scores (0-100 mm) at catheter insertion were obtained on all but two women (96 % response rate). Women scored pain with the digital technique (without using a speculum) only slightly lower (mean score 26.7, std 19.9) than with the speculum technique (mean score 28.9 std 24.7).

The response rate on the 4 week postnatal questionnaire was 67 % in the outpatient group and 73 % in the inpatient group (overall response rate of 69 %, 33/48). The majority of women (70 %) agreed or strongly agreed with the statement “The insertion of the catheter was physically uncomfortable”. Likewise, most women (58 %) agreed that they were physically uncomfortable while waiting for the catheter to work, with just over half (51 %) also agreeing that the wearing of the catheter was physically uncomfortable. More outpatient women reported being physically uncomfortable while waiting for ripening to work (68 %) than inpatient women (36 %).

Women who experienced outpatient ripening were less likely to report feeling isolated (9 % compared with 30 % of inpatients) or feeling emotionally alone (9 % vs. 36 % inpatients) during the ripening process. All outpatient women reporting that they had enough privacy during ripening (compared with only 54 % of inpatients). None of the outpatient women reporting being in noisy surroundings (compared with 27 % inpatients). Most women reported being able to start labour in a peaceful environment (91 % outpatients, 73 % inpatients). Outpatient women in general (70 %) did not have concerns about how long to remain at home. One woman was concerned about not making it back to the hospital in time.

Most women (75 %) did not report a good nights rest with the catheter, although this was more common as an inpatient (91 %) than as an outpatient (68 %). Qualitative responses suggested many women were uncomfortable with the catheter overnight, particularly when toileting, but were pleased with results in the morning. Many of the outpatient women positively commented on the opportunity to go home and be in their own environment.

Overall, women in both groups were equally satisfied with the care they received for their ripening and felt the baby was safe (91 % both groups). Approximately half of women in both groups (50 % outpatient, 45 % inpatients) agreed that they would recommend this method to others, with about a third unsure and the remaining 20 % not recommending this method of ripening.

### Acceptability of the catheter to clinicians

A total of 90 clinician questionnaires were returned (response rate of 56 % from midwives and 59 % from doctors). Over the course of the study, only seven (11 %) of midwives surveyed had inserted a balloon catheter for ripening. Of the doctors, 64 % had inserted a balloon catheter, mostly medical officers and registrars.

Scheduling and availability of staff to insert catheters was viewed as problematic by the majority of midwives (67 %) and doctors (52 %). However in terms of clinical efficacy and choice, the majority of doctors and midwives agreed or strongly agreed with the following statements; *catheters have advantages over PGEs in cervical ripening* (68 % midwives, 92 % doctors), *I am more comfortable sending home a woman overnight with a catheter than PGEs* (67 % midwives, 72 % doctors) and *eligible women should be given the option of catheter outpatient ripening* (89 % midwives, 92 % doctors). The invasiveness of balloon catheters was considered a major disadvantage over PGE_2_ gels by approximately half of all midwives (50 %) and slightly less than half of doctors (44 %).

Free text comments were overall positive, however system issues were frequently mentioned such as the need for organised staff training, especially for up skilling of midwives so that women were not kept waiting for medical practitioners and the requirement for a designated place for catheter insertions. Some midwives felt the insertion process was traumatic for certain women, such as those who were inexperienced with speculum exams or were from non-English speaking backgrounds. Midwives on the antenatal ward commented that women slept better overnight with cervical catheters compared with those who had prostaglandin ripening, as they experienced less “niggling” or contractions overnight*.*

## Discussion

This pilot study was designed to assess the clinical feasibility and acceptability to women and caregivers of outpatient balloon cervical ripening. Our findings suggest that outpatient ripening with a balloon catheter may be a viable option for appropriately screened women with low-risk, post-date pregnancies. Women who went home with a catheter had similar clinical outcomes to those who remained in hospital. There were no cases of uterine hyperstimulation or adverse outcomes associated with the catheter in either group. Although this pilot study was underpowered to demonstrate significant differences, the statistically significant finding that oxytocin use was 24 % lower in the outpatient group is of interest and would support our supposition that that women who went home were more relaxed and more likely to labour spontaneously. Although the study was not designed as a comparative analysis with prostaglandin ripening, the clinical outcomes were similar to our recent experience with the OPRA prostaglandin outpatient trial [7] with the advantage that there were no cases of hyperstimulation attributed to catheter ripening and fewer cases of fetal distress were seen in labour. This observation has been noted in specifically designed studies comparing catheter ripening with prostaglandins [3, 14, 15]. A meta analysis of 27 randomised controlled trials with 3,523 women, comparing catheters with prostaglandins revealed there was no significant difference in caesarean section, but balloon catheter ripening requires more oxytocin use [16]. The oxytocin use of 77 % in our study was consistent with the literature and was largely used for induction, rather than augmentation as would be expected with the action of non-pharmacological ripening.

A commonly used outcome measure in cervical ripening and labour induction is delivery within 24 h, although the appropriateness of this has recently been questioned [17]. In our study, only about a third of women in the two groups delivered with 24 h, consistent with other catheter studies [3, 4]. However, in a recent trial comparing two time periods for Foley catheter ripening (12 h and 24 h in situ) compared with PGE2 insert, women in the 24 h group had a significantly longer time to onset of labour as compared with the 12 h catheter group, but not in the PGE2 group. Women in both catheter groups had less side effects than the PGE2 group, suggesting a better safety profile with mechanical methods [18]. A recent large recent multicentre randomized controlled trial of 824 women demonstrated that use of a balloon catheter for ripening was just as efficacious as prostaglandins, but resulted in fewer adverse side effects, although with a longer time to delivery [14].

We did not follow a strict time-based protocol, but rather women were assessed for amniotomy the morning following the ripening process that occurred overnight. For both groups (whose catheters did not spontaneously fall out) this averaged approximately 14.5 h of having the catheter in situ. The ideal time period for keeping a catheter in situ is unknown, and recommendations range from 12–24 h, to until the catheter falls out [16, 19]. In a recent trial comparing Foley catheters with prostaglandins, catheters remained in place until expulsion or Bishop score was favourable for amniotomy up to 48 h [14]. A rigid time-based regime may not be in the best interest of women. Reasons given for the goal of delivery within 24 h of ripening (excluding high risk pregnancies) are usually cited as maternal preference [20] and the cost of hospitalization (which would self evidently not be relevant in outpatient management). However the objective of bringing about delivery within 24 h of ripening has been recently questioned [17, 21]. It is possible that women would be willing to trade off the possibility of a longer ripening to active labour duration if they were able to do this with comparable outcomes or even reduced intervention (such as syntocinon use) and avoiding additional time in hospital.

One of our primary objectives was to assess the acceptability of the catheter to women. Unlike a digital vaginal examination usually required to insert PGE2 gels, a balloon catheter insertion usually involves a speculum exam and results in a woman having a catheter protruding from her vagina and lying between her legs during the ripening process. In general, women found the catheter insertion process uncomfortable, irrespective of the digital or speculum methods, although fewer clinicians attempted the digital method. Another study assessing the two methods of catheter insertion found that the speculum group had higher pain scores as measured subjectively and objectively, however the overall satisfaction with catheter ripening was high [22]. Other studies have also found that women find the insertion uncomfortable. Henry et al. reported women were twice as likely to feel discomfort with the catheter insertion as compared with PGE2 gel insertion, however once the catheter/PGEs were inserted, they were half as likely to feel discomfort with the catheter as compared to the PGEs [4]. Another study found women rated on a visual analogue scale the double balloon catheter insertion as more uncomfortable than the single balloon, but less uncomfortable than PGE2 gel insertion [3]. The Pennell et al. study [3] also found, as did this study, that some women had difficulties in voiding when the double balloon catheter was used. However, this problem has not been reported with the single balloon catheter. It is possible that the different balloon inflation volume (two 80 ml balloons in a double catheter vs. a 30 ml balloon with a Foley catheter) contributes to the higher degree of discomfort and is more likely to cause urinary symptoms. The possibility of using less volume in the vaginal balloon of the double balloon catheter should be explored.

One of the hypotheses of outpatient ripening is that women will be more relaxed and have better sleep overnight in their home environment. It was encouraging that all but two women who were discharged home were able to remain at home until the following morning. This is in sharp contrast to the nearly 40 % of women who returned to hospital overnight with PGE2 outpatient ripening in our recent trial, mostly due to contraction onset [7]. Although women in the outpatient group reported more discomfort overnight, they also reported being more relaxed and having better sleep than women who were awaiting catheter ripening in hospital. The higher reported discomfort in the outpatient group may have been because women at home were not offered mild analgesia (inpatient women were). In the Henryetal. study, women reported significantly better sleep at home with a catheter compared with inpatient ripening with PGE2 [4]. However in this study, both groups were offered nocturnal sedation and a mild pain killer. In future studies involving outpatient cervical ripening, the option of offering mild analgesia to take home should be considered.

This was an exploratory study introducing a new paradigm of pre induction care, so we were also interested in exploring issues that could affect caregivers. While most women completed the catheter protocol (including pre and post CTG monitoring) within a timely manner, a few women had to wait up to 5 h before being seen and discharged home. This was distressing to both women and staff. A designated treatment room and improved scheduling systems for catheter insertions would greatly reduce this problem. Other systems issues noted as a concern to staff included staff training, the availability of a second person to assist and a setup package for catheter insertion. The few cases involving clinicians unable to insert the catheter was not unexpected as this was a new procedure for many. Although there were too few cases to follow over time, pain scores on insertion were anecdotally observed to decrease as operator experience increased. The majority of midwives and doctors agreed that they would be more comfortable in sending home a woman with a catheter than prostaglandins, and 90 % supported the option of offering outpatient ripening to eligible women. This supports our earlier finding with the OPRA study, that midwives supported the option of outpatient ripening [10].

Although cost were not examined, savings may be possible with outpatient ripening, as costs will be influenced by the number of overnight accommodations. In this study, outpatient women stayed at home an average of twelve and a half hours, and had a shorter length of stay. Henry et al. [4] also found that women with an outpatient catheter spent significantly less time in hospital (approximately 11 h) prior to birth, however their overall inpatient stay was not statistically shorter.

As a pilot investigation, the study is limited by the small size and lack of statistical power, which makes interpretation of small differences difficult. However, the direction of the effect is consistent with other studies and adds to the growing evidence pointing to balloon catheters as being a favourable option for cervical ripening in appropriately screened women. The response rates to questionnaires of approximately 70 % from women and 60 % from clinicians was generally good. However it could be that women and clinicians who did not have favourable views were less likely to respond. Also, the results were limited to a single metropolitan hospital, which may affect the generalizability to other facilities. The obligatory unblinded implementation of the intervention may have introduced biases in management, but this was reflective of the pragmatic nature of the trial.

## Conclusions

Results of this pilot study were encouraging from both a woman’s and clinician’s perspective. The study was able to demonstrate comparable outcomes in all clinical outcomes with the use of oxytocin being statistically significantly reduced in women allocated to the outpatient group. Clinicians supported a woman’s option for outpatient catheter ripening and were more comfortable sending women home with a catheter than with prostaglandins. The discomfort experienced by women at insertion was brief, however, reducing ongoing discomfort overnight could be investigated by offering with mild analgesia as well as by altering the balloon volume used. The catheter ripening process may be slower than pharmacological methods, but may also have better outcomes as measured by hyperstimulation and fetal distress. The possibility of cervical ripening with catheters in an outpatient setting should be explored in an adequately powered trial.

## Abbreviations

CTG: Cardiotocography
SROM: Spontaneous rupture of membranes
